# Supplementary material for: The urinary tract commensal Peptoniphilus spp. encodes a novel 17β-hydroxysteroid dehydrogenase
Source: Appl Environ Microbiol. 2026 Aug 27;92(9):e00623-26. doi: 10.1128/aem.00623-26 (PMC13599511; doi:10.1128/aem.00623-26)
Supplement: Supplementary file 1 [file aem.00623-26-s0001.docx]

**The Urinary Tract commensal *Peptoniphilus* spp*.* Encodes a Novel 17β-Hydroxysteroid Dehydrogenase**

Briawna Binion^1,2^, Saeed Ahmad^3^, Taojun Wang^1,2^, Elizabeth Tang^4^, Betsy Barnick^5,6^, Damie Olukoya^5,6^, Pauline Mbuvi^5,6^, Debapriya Dutta^5,6^, John W. Erdman Jr.^7,8^, H. Rex Gaskins^1,2,7,8^, Glen Yang^9^, Joseph Irudayaraj^1,3,5,7,10^, Jason M. Ridlon^1,2,7,8,11,12✝^

^1^Carl R. Woese Institute for Genomic Biology, Urbana, IL 61801 USA;

^2^Department of Animal Sciences, University of Illinois at Urbana-Champaign, Urbana, IL 61801

USA;

^3^Department of Bioengineering, University of Illinois at Urbana-Champaign, Urbana, IL 61801

USA;

^4^Department of Physics, University of Illinois at Urbana-Champaign, Urbana, IL, USA

^5^Carle Cancer Center, Urbana, IL, USA

^6^Department of Urology, Carle Foundation Hospital, Urbana, IL, USA.

^7^Cancer Center at Illinois, University of Illinois at Urbana-Champaign, Urbana, IL 61801 USA;

^8^Division of Nutritional Sciences, University of Illinois at Urbana-Champaign, Urbana, IL 61801

USA;

^9^Department of Urology, Carle Illinois College of Medicine, Urbana, IL

^10^Carle-Illinois College of Medicine, University of Illinois Urbana-Champaign, Urbana, IL, USA

^11^Center for Advanced Study, University of Illinois at Urbana-Champaign, Urbana, IL, U

^12^Department of Microbiology and Immunology, Virginia Commonwealth University School of

Medicine, Richmond, VA, USA;

✝Correspondence email: [jmridlon@illinois.edu](mailto:jmridlon@illinois.edu)

**Supplementary Figure 1. Steroid metabolism by *Peptoniphilus* strains.** Representative LC–MS extracted ion chromatograms showing metabolism of cortisol, 11β-hydroxyandrostenedione (11OHAD), and dehydroepiandrosterone (DHEA) by *Peptoniphilus* isolates cultured from human urine. Whole-cell incubations were performed with *Peptoniphilus genitalis* CFH36 and *Peptoniphilus obesi* CFH08, with reactions lacking bacteria included as negative controls. Chromatograms from bacterial incubations are shown alongside authentic standards for comparison. Dashed lines indicate retention times corresponding to substrate and product peaks, demonstrating strain-specific steroid metabolism by *Peptoniphilus* species.

**Supplementary Figure 2. LC–MS analysis of steroid bioconversion by rCKIMDCCC_00407.** LC–MS chromatograms illustrating the metabolism of multiple steroid substrates by rCKIMDCCC_00407. Bioconversion reactions were performed with 11β-hydroxytestosterone (11OHT), 11β-hydroxyandrostenedione (11OHAD), 11-ketotestosterone (11KT), 11-ketoandrostenedione (11KAD), dihydrotestosterone (DHT), 5α-androstenedione (5α-AD), estrone (E1), estradiol (E2), δ¹-Adrenosterone (1,4-11KAD), androstenediol (5-AL), and dehydroepiandrosterone (DHEA) using NADPH or NADP⁺ as cofactors. Each chromatogram displays the extracted ion at the indicated m/z, with substrate and product structures shown for reference. Results demonstrate cofactor-dependent interconversion of multiple androgen and estrogen substrates.

**Supplementary Figure 3.** **LC–MS analysis of steroid bioconversion by rGAN53_RS08700.** LC/MS chromatograms illustrating the metabolism of multiple steroid substrates by rGAN53_RS08700. Bioconversion reactions were performed with 11β-hydroxytestosterone (11OHT), 11β-hydroxyandrostenedione (11OHAD), 11-ketotestosterone (11KT), 11-ketoandrostenedione (11KAD), dihydrotestosterone (DHT), 5α-androstenedione (5α-AD), estrone (E1), estradiol (E2), δ¹-Adrenosterone (1,4-11KAD), androstenediol (5-AL), and dehydroepiandrosterone (DHEA) using NADPH or NADP⁺ as cofactors. Each chromatogram displays the extracted ion at the indicated m/z, with substrate and product structures shown for reference. Results demonstrate that this enzyme catalyzes interconversion of multiple androgen and estrogen substrates in both oxidative and reductive directions depending on the pyridine nucleotide cofactor supplied.

**Supplementary Table 1. Synthetic DNA constructs, primers, and sequences used in this study.** This table summarizes the constructs generated for *Anaerococcus* and *Peptoniphilus*, including organism source, DNA source type, construct description, nucleotide sequences (5′–3′), and vendor. For *Anaerococcus* (rGAN53_RS08700), a synthetic gBlock DNA fragment was used as the PCR template, and gene-specific forward and reverse primers were employed to amplify the insert for downstream applications. No native genomic DNA was used for *Anaerococcus* constructs. For Peptoniphilus (rCKIMDCCC_00407), the expression plasmid was synthesized directly by IDT using a pET-based C-His vector. All primers, gBlocks, and synthesized plasmids were obtained from IDT.

**Supplementary Figure 1**

**Supplementary Figure 2**

**Supplementary Figure 3**

**Supplementary Table 1.**
